# Preparation of pH-Sensitive Poly (N-(2-Hydroxyethyl) Acrylamide-co-acrylic Acid) Hydrogels and Their Performance

**DOI:** 10.3390/gels11040241

**Published:** 2025-03-25

**Authors:** Qiang Liu, Ge Xi, Tao Wu, Peining Li, Peng Zhan, Na Liu, Zhiping Wu

**Affiliations:** School of Chemistry and Chemical Engineering, Central South University of Forestry and Technology, Changsha 410004, China; 20221100280@csuft.edu.cn (Q.L.); 20221200222@csuft.edu.cn (G.X.); 20231200312@csuft.edu.cn (T.W.); 20231100260@csuft.edu.cn (P.L.); pzhan1982@csuft.edu.cn (P.Z.); t20232766@csuft.edu.cn (N.L.)

**Keywords:** N, N′-bis(acryloyl) cystamine, N-(2-hydroxyethyl) acrylamide, acrylic acid, pH sensitivity, mechanical properties

## Abstract

Drug-loaded hydrogels are promising for modern medicine due to their physical modifiability. However, most hydrogels suffer from poor swelling, which limits their drug encapsulation and release capabilities. In this study, Poly (N-(2-hydroxyethyl) acrylamide-co-acrylic acid) (Poly (HEAA-co-AA)) hydrogels with high swelling properties are synthesized via free radical polymerization of neutralized acrylic monomers. The effects of the material ratio and acrylic acid neutralization degree on the swelling properties of hydrogels in water are investigated, and the swelling properties of hydrogels prepared with different monomer ratios in different pH buffer solutions are systematically studied. The results show that the swelling degree is sensitive to the monomer ratio and pH. The maximum equilibrium swelling degree of the hydrogels occurs at an HEAA to AA molar ratio of 2:2, with values of 11.36 g g^−1^ at pH 1.68 and 112.79 g g^−1^ at pH 9.18. Finally, the mechanical properties of PHA hydrogels under different HEAA/AA molar ratios are investigated, showing that the mechanical properties of PHA improved compared to those of PAA. The mechanical properties of the hydrogels are best and show good stability in rheological tests when the molar ratio of HEAA to AA is 2:2. This work has major potential applications in drug carrier systems.

## 1. Introduction

Hydrogel materials have been widely used in biomedical applications due to their excellent physicochemical properties, biocompatibility, and tunable design [1]. Intelligent response hydrogels are able to change their structure and morphology based on variations in external factors such as temperature, pH, ionic strength, electromagnetic fields, and light [2]. These response behaviors provide great potential in drug delivery systems [3]. Among these, pH-responsive hydrogels have been extensively studied for drug slow-release systems, primarily due to their ability to respond to changes in the human physiological environment and the pH of the target area. This response typically results in swelling or contraction of the hydrogels, which facilitates the gradual release of drugs. The network structures of pH-responsive hydrogels generally incorporate pH-sensitive acidic or basic groups (such as carboxyl, sulfonic, or amino groups) or dynamic covalent bonds that respond to pH changes. Based on the nature of these responsive groups, pH-responsive hydrogel drug delivery systems can be classified into anionic, cationic, and zwitterionic systems [4]. As a soft material, the hydrogel was easily injured and damaged its structure when it suffered external forces during use, which resulted in the loss of its original function. Therefore, it is especially critical to prepare a hydrogel with good mechanical properties and drug-carrying capacity.

Poly (N-(2-hydroxyethyl) acrylamide) (PHEAA) hydrogels with excellent hydrophilicity can be formed from N-(2-hydroxyethyl) acrylamide (HEAA) by free radical polymerization [5]. Their hydrophilicity comes from the hydroxyl and amide groups in the side chain of PHEAA, which can form strong hydrogen bonds with the hydroxyl groups of water molecules as bond donors and acceptors. Under acidic or heating conditions, the unsaturated reactive double bonds of HEAA can copolymerize with monomers containing C=C and form a crosslinked network with excellent mechanical properties by a crosslinking reaction. Wang et al. [6] prepared a composite hydrogel that is suitable for human skin by adding gelatin extracted from cold fish skin to PHEAA chains with hydrophilic properties. Under the action of PHEAA, the elongation at the break of this hydrogel could reach 2659%.

Acrylic acid (AA) is a monomer containing carboxyl functional groups, and the pKa value of polyacrylic acid is 4.5. In [7], considerable swelling of the hydrogel occurred under specific pH conditions owing to the ionization of the carboxyl groups. Therefore, hydrogels prepared with acrylic acid (AA) generally have excellent swelling properties, but the mechanical properties of the polyacrylic acid network are poor. Zhang et al. [8] prepared polyacrylic acid hydrogels by adding a crosslinker to polyacrylic acid resin emulsion for curing. However, the tensile strength of the hydrogel was 60 kPa when the mass concentration of the crosslinker was 0.09 g mL^−1^, and low mechanical strength limits practical applications. N-(2- hydroxyethyl) acrylamide (HEAA) was incorporated into neutralized acrylic acid (AA) through copolymerization. Specifically, HEAA copolymerized with AA within the polymerization mixture, forming a crosslinked copolymer network. This incorporation enhanced the hydrogel’s mechanical properties by introducing amide and hydroxyl groups, which reinforced hydrogen bonding interactions within the network. The abundant hydroxyl groups in HEAA facilitated crosslinking with the polyacrylic acid network, significantly improving the equilibrium swelling and tensile strength of the composite hydrogel compared to polyacrylic acid alone.

Crosslinker BAC contains two acrylamide groups that efficiently participate in radical polymerization with HEAA and AA, forming a robust three-dimensional network and enhancing the mechanical stability of the hydrogel. Furthermore, the incorporation of cystamine residues improves hydrophilicity, promoting water absorption and swelling. Previous studies have demonstrated that BAC- crosslinked hydrogels exhibit excellent biocompatibility and degrade into non-toxic byproducts, making them highly suitable for biomedical applications. Bal et al. [9] synthesized a poly (methacrylic acid-acrylamide-N-hydroxyethyl acrylamide) hydrogel to investigate the effect of pH on swelling and nadolol release. In order to study the dynamic swelling process of hydrogels under different pH buffer solutions, different kinetic models such as Peppas [10] and first- order and second-order [11] were used to analyze the swelling kinetics of hydrogel samples. To evaluate the drug release kinetics at different pH values, mathematical models such as zero-order, first-order, and Higuchi models were employed to fit the drug release data.

Herein, a poly(N-(2-hydroxyethyl) acrylamide-co-acrylic acid) (PHA) hydrogel was synthesized via the reaction of a radical initiator (ammonium persulfate) and a crosslinker (N, N′- Bis(acryloyl) cystamine (BAC)). The swelling behavior of hydrogels with different monomer ratios was investigated in various pH- buffered solutions. The results demonstrated that the hydrogels exhibited significant pH sensitivity, undergoing reversible swelling-deswelling cycles in acidic and basic environments, which indicated that their swelling behavior could be effectively controlled by pH. Furthermore, the mechanical and rheological properties of the PHA hydrogels were analyzed at different HEAA/AA molar ratios, demonstrating that the mechanical strength and rheological performance of PHA improved compared to those of polyacrylic acid (PAA). These properties of PHA hydrogels provide application prospects in drug delivery, adsorption, and medical dressings.

## 2. Results and Discussion

### 2.1. Infrared Characterization of PHA Hydrogels

**Figure 1:**
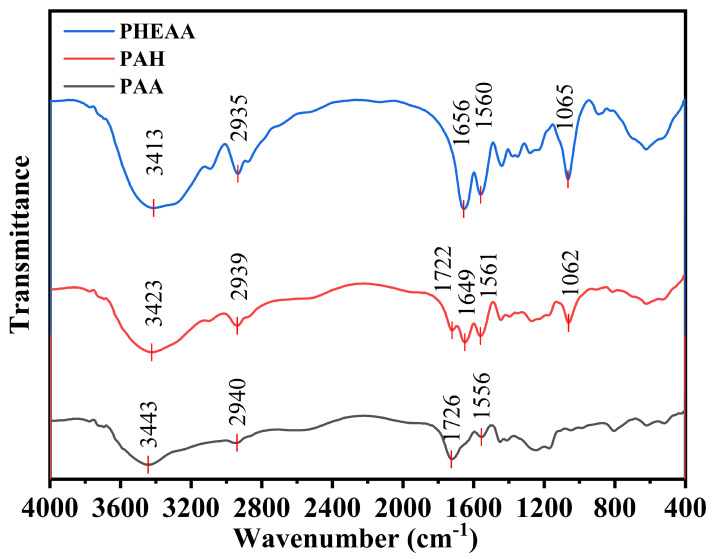
lFTIR spectra of PAA, PHEAA, and PHA hydrogels.

The FTIR spectra of PAA, PHEAA, and PHA were tested to analyze the changes in the characteristic functional groups of hydrogels (Figure 1). The peaks at 3410 cm^−1^∼ 3445 cm^−1^ are O-H stretching vibrations of PAA, PHEAA, and PHA hydrogels, and the peaks at 2935 cm^−1^∼ 2940 cm^−1^ are C-H stretching vibrations of PAA, PHEAA, and PHA hydrogels [12]. In the PAA curve, the absorption peaks of 1726 cm^−1^ and 1556 cm^−1^ represent the C=O stretching vibrations of the carboxyl group and the stretching vibrations of the carboxyl group, respectively [13]. In the PHEAA spectrum, the absorption peaks of 1656 cm^−1^, 1560 cm^−1^, and 1065 cm^−1^ represent the C=O stretching vibrations and C-N stretching vibrations of amide group, respectively [14]. In the PHA spectra, additional peaks corresponding to carboxyl C=O stretching, secondary amide C=O stretching, secondary amide C-N stretching, and tertiary amine C-N stretching were observed at 1722, 1649, 1561, and 1062 cm^−1^, indicating successful inclusion of HEAA and AA in the copolymer PHA [15].

### 2.2. Morphological Characterization of PHA Hydrogels

**Figure 2:**
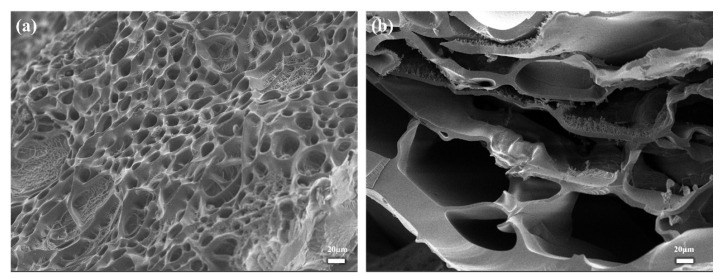
SEM images of PHA hydrogels: (**a**) SEM of PHA hydrogel immersed in pH 1.68 buffer solution; (**b**) SEM of PHA hydrogel immersed in pH 9.18 buffer solution.

The internal morphology of hydrogels immersed in buffer solutions with pH 1.68 (Figure 2a) and 9.18 (Figure 2b) were characterized by scanning electron microscopy (SEM) to visualize the effect of different pH values on the microstructure. The freeze-dried hydrogel had a three-dimensional porous structure (Figure 2), which can absorb or desorb water, providing the possibility for swelling and deswelling behavior, thus realizing an intelligent response of drug transportation [16]. The aperture of Figure 2b was larger than that of Figure 2a. This phenomenon may be attributed to the dissociation of carboxyl groups into carboxylate ions under alkaline conditions, resulting in weakened hydrogen bonding between polymer chains and enhanced electrostatic repulsion between carboxylate ions [17]. The microstructural change provided a plausible explanation for the swelling properties of hydrogels under different pH conditions and also laid the foundation for further intelligent response applications.

### 2.3. pH-Responsive Behavior of Hydrogels

#### 2.3.1. pH Response of Hydrogels with Different Monomer Ratios

**Figure 3:**
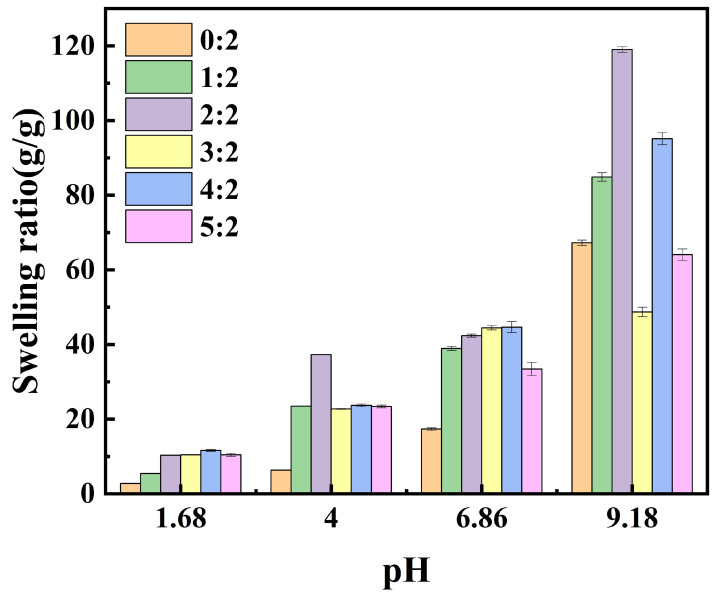
Equilibrium swelling of PHA hydrogels plotted against pH.

The equilibrium swelling degrees of hydrogels with different HEAA/AA monomer ratios as a function of pH were investigated in buffer solutions ranging from 1.68 to 9.18, as shown in Figure 3.

As illustrated in Figure 3, the equilibrium swelling degree of the hydrogels increased with pH, demonstrating notable pH sensitivity [18]. When the monomer ratio was 2:2, the equilibrium swelling degree of the hydrogels exhibited considerable variation across different pH values. This behavior differs from the optimal molar ratio observed in ultrapure water, where the swelling degree reaches 95.98 g g^−1^ when the HEAA/AA molar ratio is 2:2, suggesting a potential interplay between the ionic effect and the monomer molar ratio, a relationship that warrants further investigation. The swelling behavior of the hydrogel in ultrapure water can be found in the Supporting Information. In buffer solutions with pH values of 1.68, 4.00, 6.86, and 9.18, the equilibrium swelling degrees of the hydrogels were 10.39 g g^−1^, 18.83 g g^−1^, 48.79 g g^−1^, and 112.79 g g^−1^, respectively.

The swelling degree of PHA hydrogels in ultrapure water was much higher than that in the buffer solution. This can be explained by the absence of cations in ultrapure water, which enhances electrostatic repulsion between the -COO^−^ groups on the polymer chains, contributing to greater water absorption by the hydrogel. In the buffer solution, electrostatic interaction between cations and carboxylate ions weakens the repulsive force between the -COO^−^ groups, resulting in a significant reduction in swelling degree [19].

Under acidic conditions, the equilibrium swelling degree of all the monomers remained below 30 g g^−1^, significantly lower than that observed under alkaline conditions. Additionally, the swelling degree gradually increases with rising pH. This phenomenon is attributed to changes in the ionization state of the carboxyl groups within the hydrogel. In acidic environments, the carboxyl groups (-COOH) remain undissociated, strengthening the hydrogen bonding within the hydrogel network. This results in polymer chain contraction, thereby restricting water absorption and reducing the swelling degree. Conversely, in alkaline conditions, carboxyl groups dissociate into carboxylate ions (-COO^−^), inducing electrostatic repulsion, expanding the hydrogel network, and significantly enhancing swelling [20]. Furthermore, as pH decreases, hydrogen bonding is further reinforced, leading to a more compact network structure and an additional reduction in swelling degree.

The equilibrium swelling degrees of hydrogels for all the monomer ratios increased with pH under neutral conditions. As pH increased, the dissociation of carboxyl groups into carboxyl ions reduced the hydrogen bonding between polymer chains and enhanced the electrostatic repulsion between carboxyl ions, facilitating diffusion of the buffer solution inside the hydrogel [21].

#### 2.3.2. Effect of pH on the Swelling Behavior of Hydrogels

**Figure 4:**
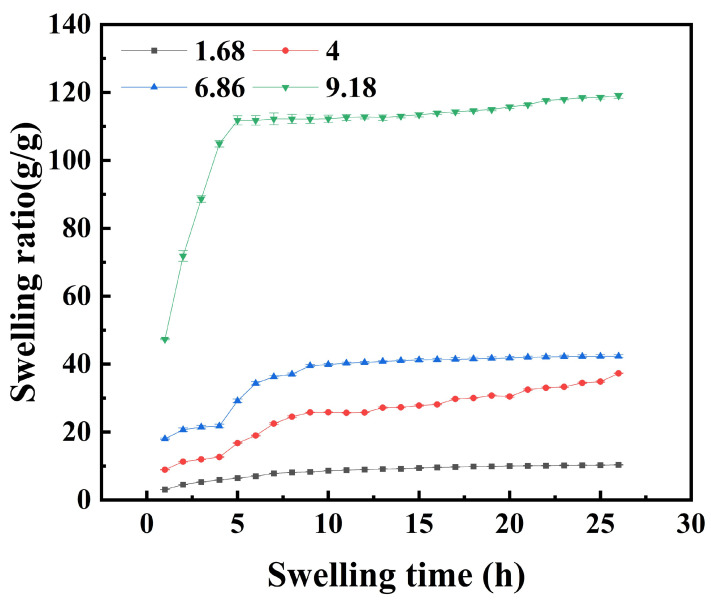
Swelling of PHA hydrogels plotted against pH.

PHA hydrogels with a monomer ratio of HEAA to AA of 2:2 were selected to investigate the pH sensitivity. At room temperature, the time-swelling curves were measured in buffer solutions with pH = 1.68, 4.00, 6.86, and 9.18, respectively, as shown in Figure 4.

The equilibrium swelling degree of the PHA hydrogels increased with ambient pH, indicating significant pH sensitivity. In addition, the swelling rate in the buffer solution at pH = 9.18 was significantly higher than in solutions with other pH values, and the swelling rate essentially increased with pH. The equilibrium swelling rate of the hydrogel in the pH buffer solution was faster than in ultrapure water, likely due to the higher ionic strength of the buffer solution. This increased ionic strength may lead to enhanced water absorption by the hydrogel.

When the pH was below the pKa value of PAA (4.5), both the swelling rate and equilibrium swelling degree increased with pH. At lower pH, the presence of external H^+^ ions limited the ionization of the -COOH group that forms hydrogen bonds inside the hydrogel, which limited the stretching and movement of the polymer chain, resulting in a low equilibrium swelling degree. As pH increased, the H^+^ concentration decreased, promoting greater dissociation of -COOH groups and enhancing electrostatic repulsion, which accelerates the swelling rate [22].

When the solution pH exceeds 4.5, the carboxylic acid groups continue to ionize into -COO^−^ anions, enhancing the electrostatic repulsion between them and expanding the hydrogel network [23]. Additionally, hydrogen bonds form between -COO^−^ groups and water molecules, further increasing the hydrogel’s water absorption capacity and swelling degree [24]. The hydrogel’s swelling performance in alkaline environments is obviously better than that in neutral and acidic environments, indicating its anion-responsive pH behavior and potential for use in drug slow-release systems.

### 2.4. Swelling Kinetics

In order to study the dynamic swelling process of hydrogels under different pH buffer solutions, different kinetic models such as Peppas, first-order, and second-order were used to analyze the swelling kinetics of hydrogel samples. The model with the highest correlation coefficient (R^2^ values explains the hydrodynamic swelling of the hydrogel. The mathematical formula of the Peppas model is interpreted as

(1) $$\ln\left(Mt/Me\right)=\ln\left(K_{1}\right)+n\ln\left(t\right),$$

(2) $$\ln\left(Me-Mt\right)=\ln\left(Me\right)-K_{2}t,$$

(3) $$\frac{t}{M_{t}}=\frac{1}{K_{3}M_{e}^{2}}+\frac{t}{M_{e}},$$

**Figure 5:**
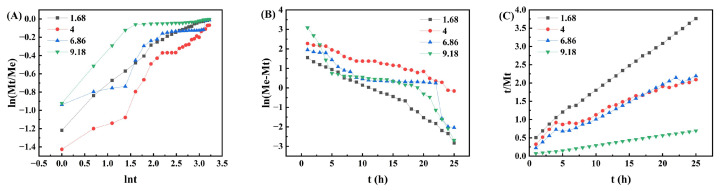
Swelling kinetics of hydrogels at different pH buffer solutions: (**A**) Peppas model fitting, illustrating the anomalous transport mechanism. (**B**) First-order kinetic model fitting, indicating a diffusion-controlled swelling process. (**C**) Second-order kinetic model fitting, demonstrating the combined effect of diffusion and relaxation mechanisms.

The swelling kinetics of PHA hydrogel with an HEAA/AA molar ratio of 2:2 were studied by fitting different kinetic models, namely the Peppas model, first-order kinetic equation, and second-order kinetic equation. The graphs obtained from these models/equations are shown in Figure 5A–C. The curve with the greatest linearity is considered to be the most suitable dynamic model. The Table 1 summarizes the swelling kinetic constants under different pH buffer solutions. The R^2^ of the second-order kinetic model is higher than that of the Peppas model and the first-order kinetic model, indicating that swelling follows the second-order dynamics in the studied pH buffer solutions (1.68, 4, 6.86, and 9.18).

### 2.5. Reversible pH Swelling of Hydrogels

**Figure 6:**
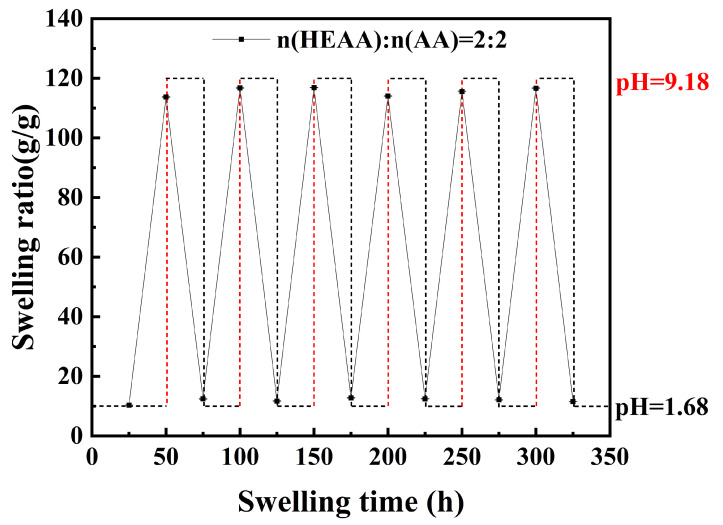
Repeated swelling and deswelling curves in pH = 1.68 and pH = 9.18 buffer solutions.

After reaching equilibrium swelling in the buffer solution at pH 1.68, the PHA hydrogel was alternately immersed in the buffer solution at pH 9.18 and pH 1.68 to evaluate its repeated swelling and deswelling behavior during the acid-base alternating cycle, as shown in Figure 6.

As shown in Figure 6, when the PHA hydrogel was transferred from a buffer solution at pH 1.68 to one at pH 9.18, its swelling degree increased from 10.19 g g^−1^ to 113.87 g g^−1^ over 25 h before decreasing to 12.63 g g^−1^ upon subsequent transfer. The hydrogels showed good recovery over six cycles, although slight fluctuations in recovery were observed after each cycle. This may be due to minor pH fluctuations caused by the diffusion and neutralization of the medium during the hydrogel’s equilibration in one pH environment before being transferred to another, resulting in slight variations in swelling recovery with increasing cycles [25].

### 2.6. Mechanical Properties of Hydrogels

**Figure 7:**
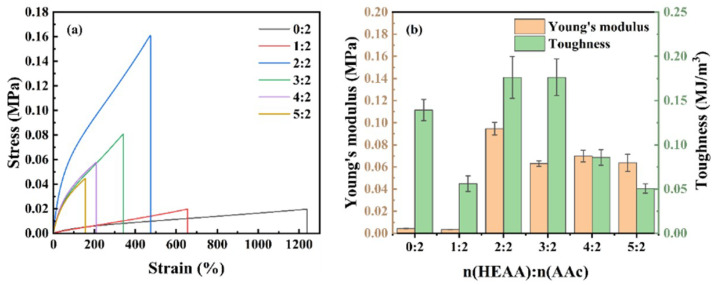
Mechanical properties of PHA hydrogels: (**a**) stress-strain curves of hydrogels with different HEAA/AA molar ratios; (**b**) modulus of elasticity and toughness of hydrogels with different HEAA/AA molar ratios.

Figure 7a presents the tensile stress-strain curves of hydrogels at different HEAA/AA molar ratios. Specific parameters can be seen in Table 2. The tensile strength and elongation at break of PHA hydrogels were 0.019 MPa and 1238.19 % when the HEAA/AA molar ratio was 0:2, and the Young’s modulus and toughness were 0.004 MPa and 0.1394 MJ m^−3^, respectively. The tensile strength of the PHA hydrogels initially increased and then decreased with rising HEAA/AA molar ratio, while the elongation at break consistently decreased. At an HEAA/AA molar ratio of 2:2, the P(HEAA-co-AA) hydrogels achieved the highest tensile strength (0.160 MPa), elongation at break (474.26%), Young’s modulus (0.095 MPa), and toughness (0.176 MJ m^−3^). The increase in HEAA content enhances the hydrogen bonding between -COOH and -CONH_2_ in the poly (HEAA-co-AA) network, which increases the number of reversible crosslinking points and improves the mechanical properties of PHA hydrogels [26]. However, a further increase in HEAA content results in higher crosslinking densities for hydrogels with a larger HEAA/AA molar ratio, restricting the free movement of polymer chains and consequently decreasing the tensile properties [27].

### 2.7. Rheological Properties of Hydrogels

**Figure 8:**
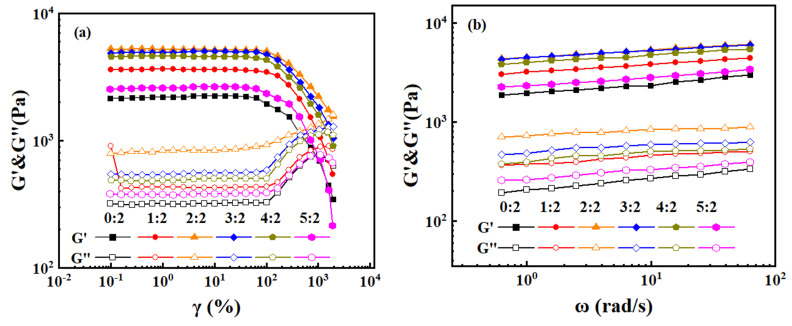
Rheological properties of PHA hydrogels: (**a**) strain amplitude scanning test at a fixed frequency of 1 Hz; (**b**) frequency scanning test at 10% fixed strain.

At room temperature, the oscillatory rheological properties of PHA hydrogels with HEAA to AA molar ratios ranging from 0:2 to 5:2 were investigated under different strains and frequencies (Figure 8). Specific parameters can be seen in Table 3. A strain-scan test was first conducted at a fixed frequency of 1 Hz in a strain range of 0.1% to 1000% to determine the linear viscoelastic region of the PHA hydrogel (Figure 8a). Subsequently, frequency scanning tests with a frequency range of 0.1 Hz to 10 Hz and a constant strain of 10% were performed to assess the effect of different monomer molar ratios on the rheological behavior of the gels (Figure 8b).

As shown in Figure 8a, the PHA hydrogels showed a strong elastic response [28]. At lower strains (<10%), G′ and G″ were independent of the applied strain and G′ was greater than G″; G′ decreases and G″ increases as the strain increases. Notably, at about 200% strain, the G′ and G″ curves intersected and the intersection point was the gel point $\gamma_{c}$. G′ was less than G″ at strains higher than 200%, indicating that the hydrogel’s mesh structure collapses at high strains. For strains below $\gamma_{c}$, the G′ value for the hydrogel with an HEAA/AA molar ratio of 0:2 was smaller than that of other molar ratios, indicating that the addition of HEAA improves the stiffness of the hydrogels.

To further elucidate the effect of the HEAA/AA molar ratio on the viscoelastic properties of PHA hydrogels, the dynamic properties of the gel material and the stability of the network structure were determined by frequency scanning tests [29], and the results obtained are shown in Figure 8b. The storage modulus G′ of the hydrogel exceeded the loss modulus G″ in all the frequency ranges, and there was no intersection between G′ and G″ [30], indicating that the hydrogel material remains in a solid-like state in the frequency range. Figure 8b. demonstrates the effect of the HEAA/AA molar ratio on the G′ and G″ properties of PHA hydrogels. The storage modulus (G′) can evaluate the degree of gel network formation, with a larger G′ value indicating higher gel strength [31]. The G′ value initially increased and then decreased as the molar ratio of HEAA/AA increased, indicating that HEAA could increase the degree of crosslinking of the hydrogels in a certain range, thereby improving the mechanical properties of the hydrogels. The peak hydrogel strength was achieved at a molar ratio of 2:2, beyond which further increases in HEAA content disrupted the crosslinked structure, resulting in a decrease in hydrogel strength.

## 3. Conclusions

In this work, a hydrogel based on N-(2-hydroxyethyl) acrylamide and acrylic acid (P(HEAA-co-AA)) was synthesized using the crosslinker N,N′-Bis(acryloyl) cystamine (BAC). The effects of the crosslinker dosage, molar ratio of monomer, initiator dosage, and neutralization degree on the swelling degree of the hydrogels were investigated. Additionally, the swelling degrees of hydrogels with different monomer molar ratios in different pH buffer solutions were analyzed and it was found that the equilibrium swelling degrees of hydrogels with all the monomer ratios increased with the increase in pH, exhibiting pronounced sensitivity of these monomer ratios to pH values.

Notably, the equilibrium swelling degree of hydrogels at a monomer ratio 2:2 fluctuated significantly under pH changes, ranging from 10.39 g g^−1^ at pH = 1.68 to 112.79 g g^−1^ at pH = 9.18. In addition, the hydrogels showed excellent pH responsiveness after undergoing six swelling and deswelling experiments with alternating cycles of acid and base. The SEM images showed that PHA hydrogels had a three-dimensional network porous structure. It was found that the pore sizes of hydrogels immersed in buffer solutions with pH values of 1.68 and 9.18 were extremely different, which was consistent with the differences in the equilibrium swelling degrees of hydrogels in different pH environments. Increases in the HEAA/AA molar ratio also affected the mechanical and rheological properties of P(HEAA-co-AA) hydrogels. The tensile strength of the hydrogels gradually increased with the HEAA/AA molar ratio and reached the maximum at an HEAA/AA molar ratio of 2:2, which substantially improved compared to that of PAA. This indicates that the incorporation of HEAA effectively improves the mechanical properties of the hydrogels. In addition, the hydrogels showed better stability in a certain frequency range.

## 4. Materials and Methods

### 4.1. Materials

N-(2-hydroxyethyl) acrylamide (HEAA), N,N′-Bis(acryloyl) Cystamine (BAC), Ammonium persulfate (APS), N,N,N′,N′- Tetramethylethylenediamine (TEMED), potassium tetraoxalate dihydrate, and potassium bitartrate are obtained from Shanghai Macklin Biochemical Technology Co., Ltd., Shanghai, China. Acrylic acid (AA), potassium hydrogen phthalate, disodium hydrogen phosphate, and potassium dihydrogen phosphate are purchased from Sinopharm Group Chemical Reagent Co., Ltd., Shanghai, China. Sodium tetraborate comes from Hunan Huihong Reagent Co., Ltd., Changsha, China., and sodium hydroxide comes from Tianjin Damao Chemical Reagent Factory, Tianjin, China. Sodium tetraborate decahydrate (AR) comes from Hunan Huihong Reagent Co., Ltd., and sodium hydroxide (AR) comes from Tianjin Damao Chemical Reagent Factory. All reagents were analytical reagents and without further purification.

### 4.2. Preparation of Poly (N-(2-Hydroxyethyl) Acrylamide-acrylic acid) (PHA) Hydrogel

A total of 0.2042 g of sodium hydroxide was dissolved in 1.5 mL of deionized water. Under ice bath conditions, 0.8 mL of acrylic acid (AA) was gradually added to the sodium hydroxide solution, followed by 1.2 mL of N-(2-hydroxyethyl) acrylamide (HEAA). The total monomer volume was maintained at 2 mL, with the respective volumes of HEAA and AA adjusted based on different HEAA-to-AA molar ratios. Subsequently, 2 mL of 1.25 wt% ammonium persulfate (APS), 1 mL of 1.6 mmol/L N, N′-bis(acryloyl) cystamine (BAC), and 50 μL of N,N,N′,N′-tetramethylethylenediamine (TEMED) were added under a nitrogen atmosphere. The mixture was then solidified into a hydrogel in an oven at 70 °C. To remove unreacted components, the hydrogel was repeatedly soaked in ultrapure water. For both PHEAA and PAA hydrogels, the total monomer volume remained 2 mL, with all other synthesis conditions identical to those of PHA hydrogels. Finally, all hydrogels were freeze-dried to a constant weight.

### 4.3. Characterization of PHA Hydrogels

The FTIR spectra of PHEAA, PAA, and PHA hydrogels were acquired with a Fourier Transform infrared spectrometer (Thermo Fisher Scientific Nicolet iS20, Waltham, MA, USA). Each sample was scanned 32 times with a scanning range of 400 to 4000 cm^−1^ and a resolution set to 4 cm^−1^. A scanning electron microscope (TESCAN MIRA LMS, Brno, Czech Republic) was used to observe the morphology of the hydrogels at an accelerated electron energy of 5.0 kV. The hydrogels were freeze-dried and sputtered with gold prior to SEM observation.

### 4.4. Testing of Hydrogel Swelling Properties

#### 4.4.1. Determination of Swelling Degree of Hydrogels

The dried gel was immersed in ultrapure water, the mass was weighed at intervals, and the swelling degree of the hydrogels was calculated by gravimetric method. The swelling degree (SD, g g^−1^) was calculated according to the following equation:

(4) $$Swelling\;Degree\;\left(SD\right)=\frac{\left(m_{t}-m_{0}\right)}{m_{0}},$$

where $m_{t}$ is the weight of hydrogel measured at different time points, and $m_{0}$ is the mass of the dry gel.

#### 4.4.2. pH Sensitivity Testing of Hydrogels

Buffer solutions of different pH values were prepared using specific reagents. A pH 1.68 buffer was prepared by dissolving 12.61 g of potassium tetraoxalate (KH_3_C_4_O_8_·2H_2_O) in 1 L of deionized water. A pH 4.00 buffer was obtained by dissolving 10.12 g of potassium hydrogen phthalate (HOOCC_6_H_4_COOK) in 1 L of deionized water. A phosphate buffer solution (pH 6.86) was prepared using disodium hydrogen phosphate (Na_2_HPO_4_) and potassium dihydrogen phosphate (KH_2_PO_4_). Specifically, 3.387 g of Na_2_HPO_4_ and 3.402 g of KH_2_PO_4_ were dissolved in 1 L of deionized water, ensuring complete dissolution by magnetic stirring. For the pH 9.18 buffer, 3.8 g of sodium tetraborate (Na_2_B_4_O_7_) was dissolved in 1 L of deionized water. Dry samples were placed in buffer solutions with different pH values (pH values were 1.68, 4.00, 6.86, and 9.18, respectively). At regular intervals, the hydrogel was taken out from the solution and excess water was removed, and the equilibrium swelling degree was measured until the hydrogel reached a constant weight. According to Equation (4), the swelling degrees of hydrogels in buffer solutions with different pH values were calculated.

The hydrogel was first swollen to an equilibrium state in a buffer solution with a pH of 1.68 and then transferred to a buffer solution with a pH of 9.18, alternating swelling and deswelling processes. This process was repeated six times until the hydrogel was in a swelling/deswelling equilibrium. Use Equation (4) to calculate the swelling degree for each swelling/deswelling equilibrium.

### 4.5. Testing of Mechanical Properties of Hydrogels

The mechanical properties of hydrogels were characterized using an HY-0350 tensile elastic modulus tester(Shanghai Hengyi Testing Instruments Co., Ltd., Shanghai, China). The hydrogel used for the test is derived from a freshly cured hydrogel. The size of testing strips was 30 mm × 1.5 mm (width × thickness). Uniaxial tensile tests were performed at a speed of 20 mm min^−1^. The specific parameters are defined as follows: The tensile modulus was determined by the slope of the stress-strain curve in the linear strain range of 0∼2%. The tensile strength was expressed by the stress value at the breaking point, and the elongation at the break was expressed by the strain value at the breaking point. Toughness was calculated by the integral area between the stress-strain curve and the X-axis. All data of mechanical properties were the average of valid data for at least three samples to ensure the reliability of the experimental results.

### 4.6. Rheological Testing of Hydrogels

Rheological analysis of hydrogels was performed using a rotational rheometer DHR-2 (TA, New Castle, DE, USA). The hydrogel used for the test is derived from a freshly cured hydrogel. The disc-shaped hydrogel sample was prepared in a 25 mm diameter sample bottle, resulting in a hydrogel with a 25 mm diameter and 2 mm thickness. The disc-shaped hydrogel sample was fixed between two parallel plates of the rheometer (40 mm in diameter and 2 mm in thickness) and the rheological properties of the hydrogels were evaluated by the following two modes: Dynamic strain sweeps were first performed at an oscillation frequency of 1 Hz over a strain range of 0.1∼1000% to determine the linear viscoelastic region (the region where the modulus is independent of the strain amplitude) by the change in storage modulus (G′) and loss modulus (G″). The storage modulus (G′) and loss modulus (G″) were measured in the angular frequency range from 0.1 Hz to 10 Hz at a fixed 10% oscillation strain. 

## Figures and Tables

**Table 1:** The swelling kinetic constants at different pH.

	Peppas Model			First-Order Model		Second-Order Model	
pH	R^2^	n	K_1_	R^2^	K_2_	R^2^	K_3_
1.68	0.9641	0.355	0.343	0.9802	0.166	0.9986	0.035
4	0.9595	0.45	0.225	0.954	0.092	0.9744	0.01
6.86	0.8883	0.299	0.388	0.748	0.123	0.9859	0.028
9.18	0.6899	0.203	0.563	0.8167	0.161	0.9987	0.028

**Table 2:** Mechanical strength of hydrogels with different monomer molar ratios.

HEAA: AA Molar Ratio	Tensile Strength (MPa)	Elongation at Break (%)	Young’s Modulus (MPa)	Toughness (MJ/m^3^)
0:2	0.019	1238.19	0.004	0.139
1:2	0.020	655.73	0.003	0.056
2:2	0.160	474.26	0.095	0.176
3:2	0.081	341.76	0.063	0.176
4:2	0.058	209.19	0.070	0.086
5:2	0.045	156.60	0.064	0.051

**Table 3:** Variation trend of G′ and G″ under different HEAA/AA ratios.

HEAA/AA Ratios	G′ (Pa)	G″ (Pa)
0:2	346∼2248	315∼768
1:2	544∼3637	424∼904
2:2	1738∼5257	791∼1584
3:2	1043∼5021	541∼1285
4:2	905∼4617	480∼1162
5:2	214∼2645	378∼776

## Data Availability

The original contributions of this study are included in the article. Further inquiries can be directed to the corresponding author.

## Supplementary Materials

The following supporting information can be downloaded at https://www.mdpi.com/article/10.3390/gels11040241/s1, Refs. [32,33,34,35,36,37,38] are cited in Supplementary Materials. Figure S1. Influence of dosage of crosslinker on swelling degree of PHA hydrogels. Figure S2. Effect of monomer ratios on the swelling degree of PHA hydrogels. Figure S3. Influence of dosage of initiator on the swelling degree of PHA hydrogels. Figure S4. Effect of different degrees of neutralization on solubility of PHA hydrogels.

## References

[1] Zagórska-Dziok M., Sobczak M. (2020). Hydrogel-based active substance release systems for cosmetology and dermatology application: A review. Pharmaceutics.

[2] Bordbar-Khiabani A., Gasik M. (2022). Smart hydrogels for advanced drug delivery systems. Int. J. Mol. Sci..

[3] Liu T., Du Y., Yan Y., Song S., Qi J., Xia X., Hu X., Chen Q., Liu J., Zeng X. (2023). pH-responsive dual-functional hydrogel integrating localized delivery and anti-cancer activities for highly effective therapy in PDX of OSCC. Mater. Today.

[4] Zixuan C., Bin Z., Liyang J., Yunyi L., Guohe X., Jingjun M. (2019). Intelligent-Responsive Hydrogels-Based Controlled Drug Release Systems and Its Applications. Prog. Chem..

[5] Zhang J., Xiao S., Shen M., Sun L., Chen F., Fan P., Zhong M., Yang J. (2016). Aqueous lubrication of poly (N-hydroxyethyl acrylamide) brushes: A strategy for their enhanced load bearing capacity and wear resistance. RSC Adv..

[6] Wang S., Wang F., Shi K., Yuan J., Sun W., Yang J., Chen Y., Zhang D., Che L. (2022). Osteichthyes skin-inspired tough and sticky composite hydrogels for dynamic adhesive dressings. Compos. Part B Eng..

[7] De Breuck J., Streiber M., Ringleb M., Schröder D., Herzog N., Schubert U.S., Zechel S., Traeger A., Leiske M.N. (2024). Amino-Acid-Derived Anionic Polyacrylamides with Tailored Hydrophobicity–Physicochemical Properties and Cellular Interactions. ACS Polym. Au.

[8] Zhang J., Qu D., Wang S., Qi S., Zuo H. (2024). Structure, Property Optimization, and Adsorption Properties of N, N^′^-methylenebisacrylamide Cross-Linked Polyacrylic Acid Hydrogels under Different Curing Conditions. Polymers.

[9] Bal A., Özkahraman B., Özbaş Z. (2016). Preparation and characterization of p H responsive poly (methacrylic acid-acrylamide-N-hydroxyethyl acrylamide) hydrogels for drug delivery systems. J. Appl. Polym. Sci..

[10] Awasthi S., Singhal R. (2015). Mathematical modeling for the prediction of the overall swelling profile from poly (AM-co-AA-co-HEA) hydrogels: Effect of glycidyl methacrylate and ammonium per sulphate. Int. J. Plast. Technol..

[11] Zagni C., Dattilo S., Mecca T., Gugliuzzo C., Scamporrino A.A., Privitera V., Puglisi R., Carroccio S.C. (2022). Single and dual polymeric sponges for emerging pollutants removal. Eur. Polym. J..

[12] Didehban K., Hayasi M., Kermajani F. (2017). Removal of anionic dyes from aqueous solutions using polyacrylamide and polyacrylic acid hydrogels. Korean J. Chem. Eng..

[13] Barbani N., Bertoni F., Ciardelli G., Cristallini C., Silvestri D., Coluccio M., Giusti P. (2005). Bioartificial materials based on blends of dextran and poly (vinyl alcohol-co-acrylic acid). Eur. Polym. J..

[14] Ma M., Li Y., Wang D., Li J., Ye H., Du D., Chi R., Han Q. (2024). Highly efficient and sustainable polyacrylic acid-polyacrylamide double-network hydrogels prepared by cross-linking of waste residues for heavy metals ions removal. J. Environ. Chem. Eng..

[15] Zhao B., Jiang H., Lin Z., Xu S., Xie J., Zhang A. (2019). Preparation of acrylamide/acrylic acid cellulose hydrogels for the adsorption of heavy metal ions. Carbohydr. Polym..

[16] Wang X., Wang Y., He S., Hou H., Hao C. (2018). Ultrasonic-assisted synthesis of superabsorbent hydrogels based on sodium lignosulfonate and their adsorption properties for Ni^2+^. Ultrason. Sonochem..

[17] Rodríguez-Loredo N.A., Ovando-Medina V.M., Pérez E., Corona-Rivera M.A., Cervantes-González E., Antonio-Carmona I.D., Ramos-Torres C.J. (2023). Preparation of poly (acrylic acid)/linseed mucilage/chitosan hydrogel for ketorolac release. J. Vinyl Addit. Technol..

[18] Markovic M.D., Panic V.V., Seslija S.I., Spasojevic P.M., Ugrinovic V.D., Boskovic-Vragolovic N.M., Pjanovic R.V. (2020). Modification of hydrophilic polymer network to design a carrier for a poorly water-soluble substance. Polym. Eng. Sci..

[19] Wang Y., He G., Li Z., Hua J., Wu M., Gong J., Zhang J., Ban L.t., Huang L. (2018). Novel biological hydrogel: Swelling behaviors study in salt solutions with different ionic valence number. Polymers.

[20] Corona Rivera M.A., Cisneros Covarrubias C.A., Fernández Escamilla V.V.A., Mendizábal Mijares E., Pérez López J.E. (2022). Synthesis and characterization of pH-responsive water-dispersed nanohydrogels of cross-linked polyacrylamide-co-polyacrylic acid. Polym. Eng. Sci..

[21] Thakur S., Arotiba O.A. (2018). Synthesis, swelling and adsorption studies of a pH-responsive sodium alginate–poly (acrylic acid) superabsorbent hydrogel. Polym. Bull..

[22] Pourjavadi A., Kurdtabar M. (2007). Collagen-based highly porous hydrogel without any porogen: Synthesis and characteristics. Eur. Polym. J..

[23] Nesrinne S., Djamel A. (2017). Synthesis, characterization and rheological behavior of pH sensitive poly (acrylamide-co-acrylic acid) hydrogels. Arab. J. Chem..

[24] Li Q., Ma W., Ma H., Liu H., Shang H., Qiao N., Sun X. (2024). pH-responsive biomass-based hydrogels for 5-fu delivery: Investigating the influential role of itaconic acid content on structural characteristic, swelling behavior, and drug loading capacity. Mater. Chem. Phys..

[25] Wang W., Wang A. (2010). Synthesis and swelling properties of pH-sensitive semi-IPN superabsorbent hydrogels based on sodium alginate-g-poly (sodium acrylate) and polyvinylpyrrolidone. Carbohydr. Polym..

[26] Jing Z., Xu A., Liang Y.Q., Zhang Z., Yu C., Hong P., Li Y. (2019). Biodegradable poly (acrylic acid-co-acrylamide)/poly (vinyl alcohol) double network hydrogels with tunable mechanics and high self-healing performance. Polymers.

[27] Wang J., Liu Y., Wang S., Liu X., Chen Y., Qi P., Liu X. (2021). Molybdenum disulfide enhanced polyacrylamide-acrylic acid-Fe3+ ionic conductive hydrogel with high mechanical properties and anti-fatigue abilities as strain sensors. Colloids Surfaces A Physicochem. Eng. Asp..

[28] Jing Z., Zhang Q., Liang Y.Q., Zhang Z., Hong P., Li Y. (2019). Synthesis of poly (acrylic acid)–Fe^3+^/gelatin/poly (vinyl alcohol) triple-network supramolecular hydrogels with high toughness, high strength and self-healing properties. Polym. Int..

[29] Hu X., Feng L., Wei W., Xie A., Wang S., Zhang J., Dong W. (2014). Synthesis and characterization of a novel semi-IPN hydrogel based on Salecan and poly (N,N-dimethylacrylamide-co-2-hydroxyethyl methacrylate). Carbohydr. Polym..

[30] Shin J., Choi S., Kim J.H., Cho J.H., Jin Y., Kim S., Min S., Kim S.K., Choi D., Cho S.W. (2019). Tissue tapes—phenolic hyaluronic acid hydrogel patches for off-the-shelf therapy. Adv. Funct. Mater..

[31] Mandal B.B., Kapoor S., Kundu S.C. (2009). Silk fibroin/polyacrylamide semi-interpenetrating network hydrogels for controlled drug release. Biomaterials.

[32] Zhou X., Ma Y., Wang N., Lei Z. (2024). Facile synthesis of an acrylic acid-co-2-acrylamide-2-methylpropanesulfonic acid copolymer/polyvinylpyrrolidone semi-IPN hydrogel with excellent swelling and anti-leakage properties. Mater. Today Commun..

[33] Olad A., Zebhi H., Salari D., Mirmohseni A., Reyhanitabar A. (2018). A promising porous polymer-nanoclay hydrogel nanocomposite as water reservoir material: Synthesis and kinetic study. J. Porous Mater..

[34] Li Y., Li X., Chen C., Zhao D., Su Z., Ma G., Yu R. (2016). A rapid, non-invasive and non-destructive method for studying swelling behavior and microstructure variations of hydrogels. Carbohydr. Polym..

[35] Chen T., Liu H., Dong C., An Y., Liu J., Li J., Li X., Si C., Zhang M. (2020). Synthesis and characterization of temperature/pH dual sensitive hemicellulose-based hydrogels from eucalyptus APMP waste liquor. Carbohydr. Polym..

[36] Ramazani-Harandi M., Zohuriaan-Mehr M., Yousefi A., Ershad-Langroudi A., Kabiri K. (2009). Effects of structural variables on AUL and rheological behavior of SAP gels. J. Appl. Polym. Sci..

[37] Chen M., Shen Y., Xu L., Xiang G., Ni Z. (2020). Synthesis of a super-absorbent nanocomposite hydrogel based on vinyl hybrid silica nanospheres and its properties. RSC Adv..

[38] Liu X., Cheng S., Zhao J., Qiu X., Zhang W., Ma G., Lei Z. (2021). Preparation and anti-leakage properties of sesbania gum-grafted copolymers. J. Appl. Polym. Sci..

