# Supplementary material for: Preparation of pH-Sensitive Poly (N-(2-Hydroxyethyl) Acrylamide-co-acrylic Acid) Hydrogels and Their Performance
Source: Gels. 2025 Mar 25;11(4):241. doi: 10.3390/gels11040241 (PMC12027044; doi:10.3390/gels11040241)
Supplement: Supplementary file 1 [file gels-11-00241-s001.zip › gels-3497041-supplementary.pdf]

Preparation of pH-sensitive poly (N-(2-hydroxyethyl) acrylamide -co-acrylic acid) and its performance

*Qiang Liu, Ge Xi, Tao Wu, Peining Li, Peng Zan, Na Liu and Zhiping Wu**
School of Chemistry and Chemical Engineering, Central South University of Forestry and Technology, 410004, China
E-mail: wuzhiping02@163.com

2. Experimental section

2.1. Materials

N-(2-hydroxyethyl) acrylamide (HEAA), N, N'-Bis(acryloyl) cystamine (BAC), Ammonium persulfate (APS), N, N, N', N'-Tetramethylethylenediamine (TEMED), Potassium tetraoxalate dihydrate, and Potassium bitartrate are obtained from Macklin reagent. Acrylic acid (AA), potassium hydrogen phthalate, disodium hydrogen phosphate and potassium dihydrogen phosphate are purchased from Sinopharm Chemical Reagent Co., Ltd. Sodium tetraborate comes from Hunan Huihong Reagent Co., Ltd., sodium hydroxide comes from Tianjin Damao Chemical Reagent Factory. Sodium tetraborate decahydrate (AR) comes from Hunan Huihong Reagent Co., Ltd., and sodium hydroxide (AR) comes from Tianjin Damao Chemical Reagent Factory. All reagents were analytical reagent and without its further purification.

2.2. Preparation of poly (N -(2-hydroxyethyl) acrylamide-acrylic) (PHA) hydrogel

Acrylic acid (AA) was charged into a 50 ml sample bottle bathed in ice, sodium hydroxide of the corresponding neutralization degree was dissolved in deionized water and added dropwisely under continuous shaking. After 5 min of reaction, different amounts of *N*-(2-hydroxyethyl) acrylamide (HEAA) were added according to predetermined molar ratios of HEAA to AA (1:2, 2:2, 3:2, 4:2, and 5:2). Crosslinker *N, N'*-Bis(acryloyl) cystamine (BAC) and activator *N, N, N', N'*-tetramethylethylenediamine (TEMED) were added in above solution and oscillated until completely dissolved. Nitrogen and APS were added to the bottle and sealed at

70 °C for one hour. PHA was obtained by heating, using ultra-pure water to remove the reaction residue. PHEAA and PAA were prepared by adding single HEAA or AA in the same reaction condition. The hydrogels dried in a freeze dryer to a constant weight.

2.3. Characterization of PHA hydrogels

The FTIR spectra of PHEAA, PAA, and PHA hydrogels were acquired with a Fourier Transform infrared spectrometer (Thermo Fisher Scientific Nicolet iS20). Each sample was scanned 32 times with a scanning range of 400 to 4000 cm^{-1} and a resolution set to 4 cm^{-1}. A scanning electron microscope (TESCAN MIRA LMS) was used to observe the morphology of the hydrogels at an accelerated electron energy of 5.0 kV. The hydrogels were freeze-dried and sputtered with gold prior to SEM observation.

2.4. Testing of hydrogel swelling properties

2.4.1. Determination of swelling degree of hydrogels

The dried gel was immersed in ultra-pure water, the mass was weighed at intervals, and the swelling degree of the hydrogel was calculated by gravimetric method. The swelling degree (SD, g g^{-1}) was calculated according to the following equation:

$$\text{Swelling Degree(SD)} = \frac{(m_t - m_0)}{m_0} \quad (1)$$

where m_t is the weight of hydrogel measured at different time points, and m_0 is the mass of the dry gel.

2.4.2. pH sensitivity testing of hydrogels

Dry samples were placed in buffer solutions with different pH values (pH values were 1.68, 4.00, 6.86 and 9.18, respectively). At regular intervals, the hydrogel was taken out from the solution and excess water was removed, the equilibrium swelling degree was measured until the hydrogel reached a constant weight. According to equation (1), the swelling degree of hydrogel in buffer solution with different pH values was calculated.

2.4.3. Swelling - Deswelling Test for Hydrogels

The hydrogel was first swollen to an equilibrium state in a buffer solution with a pH of 1.68, and then transferred to a buffer solution with a pH of 9.18, alternating swelling and deswelling processes. This process was repeated six times until the

hydrogel was in a swelling/deswelling equilibrium. Use equation (1) to calculate the swelling degree for each swelling/deswelling equilibrium.

2.5. Testing of mechanical properties of hydrogels

The mechanical properties of hydrogels were characterized using a HY-0350 tensile elastic modulus tester. The size of testing strips was 30 mm × 1.5 mm (width × thickness). Uniaxial tensile tests were performed at a speed of 20 mm min^{-1}. The specific parameters are defined as follows: The tensile modulus was determined by the slope of the stress-strain curve in the linear strain range of 0~2%. The tensile strength was expressed by the stress value at the breaking point, and the elongation at the break was expressed by the strain value at the breaking point. Toughness was calculated by the integral area between the stress-strain curve and the X-axis. All mechanical properties data were the average of valid data for at least three samples to ensure the reliability of the experimental results.

2.6. Rheological testing of hydrogels

Rheological analysis of hydrogels was performed using a rotational rheometer DHR-2. The disc-shaped hydrogel sample was fixed between two parallel plates of the rheometer (40 mm in diameter and 2 mm in thickness) and the rheological properties of the hydrogels was evaluated by the following two modes: Dynamic strain sweeps were first performed at an oscillation frequency of 1 Hz over a strain range of 0.1%-1000% to determine the linear viscoelastic region (the region where the modulus is independent of the strain amplitude) by the change in storage modulus (G') and loss modulus (G"); The storage modulus (G ') and loss modulus (G ") were measured in the angular frequency range from 0.1Hz to 10Hz at a fixed 10% oscillation strain.

3. Results and discussion

3.1. Swelling properties of hydrogels

3.1.1. Effect of crosslinker dosage on the swelling degree of hydrogels

The dosage of crosslinker directly affected the crosslinking degree of hydrogel and its network structure. The effect of crosslinker dosage on the swelling degree of hydrogels were illustrated in **Figure S1**.

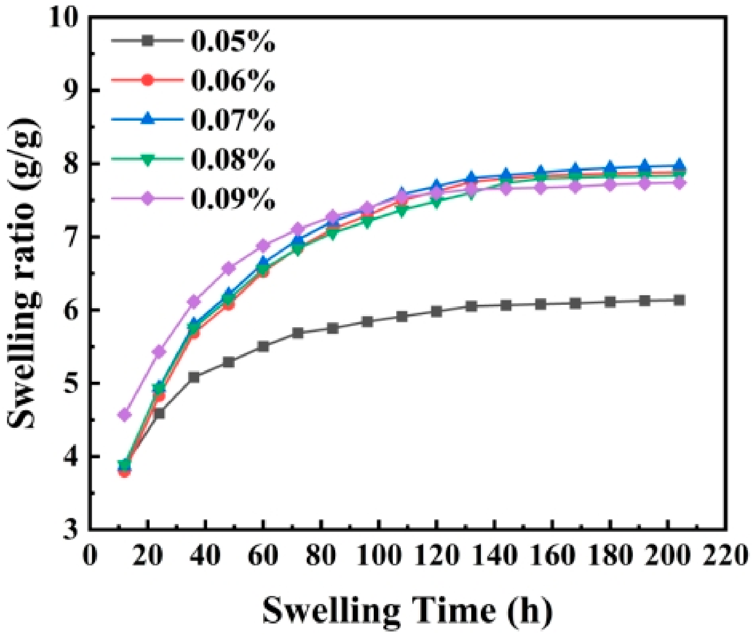

Figure S1 Influence of dosage of crosslinker on swelling degree of PHA hydrogel

The equilibrium swelling degree of hydrogel was the lowest when the dosage of crosslinker was 0.05%. At a lower dosage of BAC, there are fewer cross-linking points between polymer chains, and the low cross-linking density leads to the formation of fewer three-dimensional network structures, resulting in lower swelling degree.[1] The number of cross-linking points increased with dosage of the crosslinker, the three-dimensional network structure was enhanced and the equilibrium swelling degree of the hydrogel increased. The equilibrium swelling of PHA hydrogel reached 7.94 g g^{-1} when the crosslinker dosage reached 0.07 wt%. However, the equilibrium swelling degree decreased slightly when the dosage of crosslinker was 0.09%. Which can be explained that too much high cross-linking density shortened the length of polymer chains between adjacent cross-linking points, then the extensibility of the polymer network was limited, so the equilibrium swelling degree was reduced.[2]

3.1.2. Effect of monomer molar ratio on the swelling of hydrogels

The effect of monomer molar ratio on the swelling of hydrogels were illustrated in **Figure S2**.

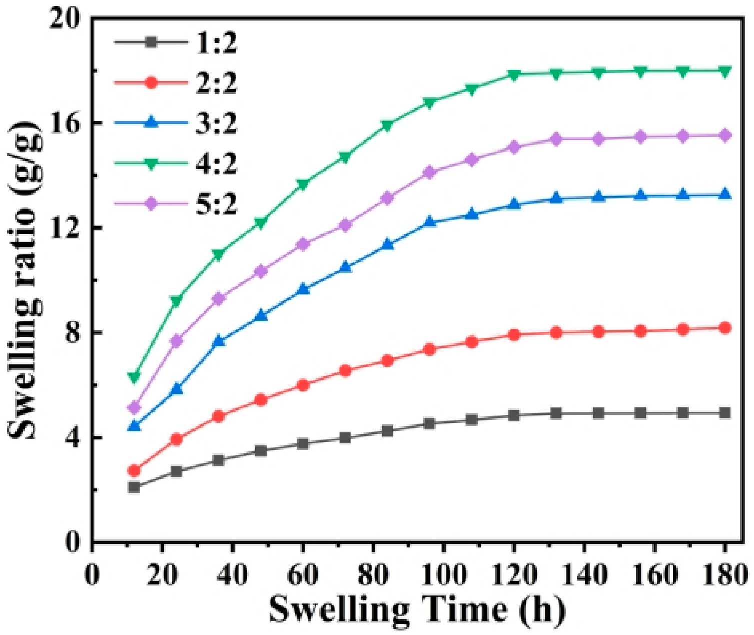

Figure S2 Effect of monomer ratios on the swelling degree of PHA hydrogels

As can be seen from Figure S2, the equilibrium swelling degree of the hydrogel was positively correlated with the molar ratio of HEAA to AA when the mole ratio was less than 4:2. It can be explained that HEAA is non-ionic monomer, its hydrophilic group $-CONH_2$ and the anionic hydrophilic group –COONa of acrylic acid has the cooperative effect, which lead to an increase in equilibrium swelling degree.[3] The equilibrium swelling of the hydrogel reached a maximum value of 17.39 g g^{-1} when the molar ratio of HEAA to AA was 4:2. But equilibrium swelling degree decreased when the molar ratio of HEAA to AA was 5:2. The reason is that the hydrophilicity of $-CONH_2$ of HEAA is worse than that of -COONa and -COOH of acrylic acid, at the same time, the Na^+ concentration difference between the inside and outside of the network is reduced and the osmotic pressure is lowered, So too much high molar ratio of HEAA to AA will lead to the decrease of equilibrium swelling degree.

3.1.3. Effect of initiator dosage on the swelling degree of hydrogels

The dosage of initiator usually affects the degree of crosslinking of hydrogels and the length of polymer chains, thus affecting their network structure. **Figure S3** showed the swelling degree of the hydrogel increased with initiator dosage.

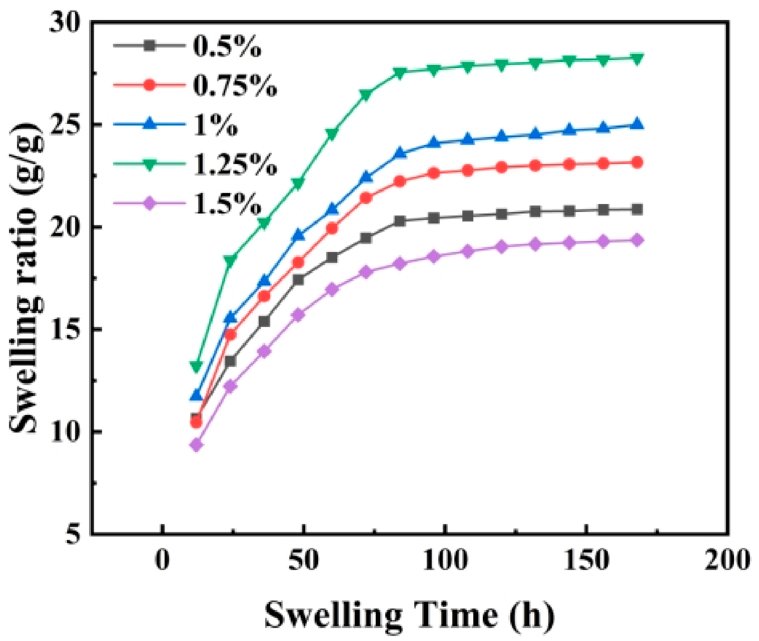

Figure S3 Influence of dosage of initiator on the swelling degree of PHA hydrogel

The maximum swelling degree of 29.55 g g^{-1} was reached when the initiator dosage was 1.25 wt%. This can be explained by the facts that more initiator can generate more free radicals, which accelerates the polymerization reaction and enhances the swelling degree.[4] Nevertheless, further increasing initiator dosage led to a decrease in swelling degree. The reason is that the chain termination reaction becomes more pronounced with higher initiator dosage, resulting in shorter polymer chains. Additionally, copolymerized monomers may undergo burst polymerization or self-polymerization, which reduces the polymer's molecular weight and disrupts the network structure, ultimately lowering the swelling degree.[5]

3.1.4. Effect of degrees of neutralization on the swelling rate of hydrogels

The effect of degrees of neutralization on the swelling of hydrogels were illustrated in **Figure S4**. The neutralization of acrylic acid can not only increase the swelling rate, but also reduce the swelling equilibrium time.

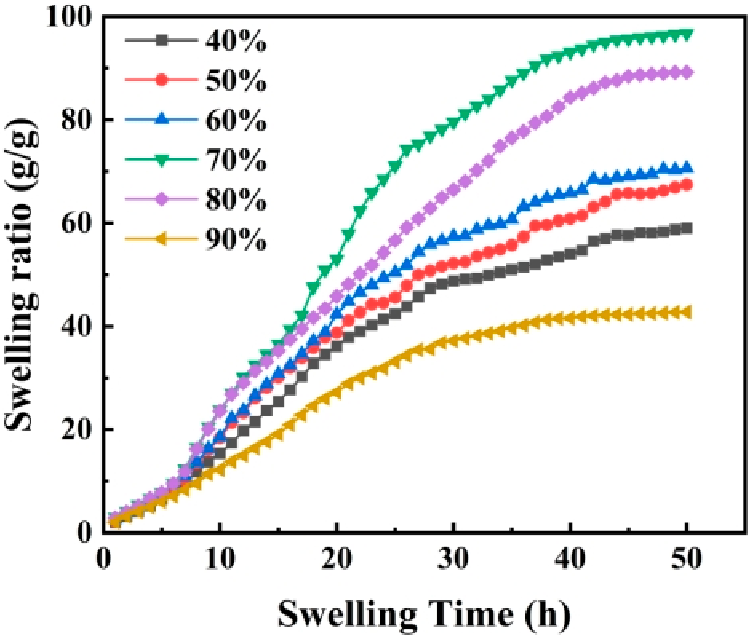

Figure S4 Effect of different degrees of neutralization on solubility of PHA hydrogels

The equilibrium swelling degree of PHA hydrogels increased gradually with the degree of acrylic neutralization. It reached a maximum of 95.98 g g^{-1} when the neutralization degree was 70%. Nevertheless, the equilibrium swelling degree decreased to 45.01 g g^{-1} as the neutralization degree increased to 90%. The reason for this phenomenon is as follows: when the neutralization degree is less than 70%, the ionic hydrophilic effect and osmotic pressure difference increase, while the electrostatic repulsion among negatively charged carboxylate groups expands the polymer network.[6] However, excessive neutralization generates more sodium ions within the polymer network, which screen the negative charges of COO^{-} groups, reducing electrostatic repulsion and decreasing the equilibrium swelling degree. [7]

According to the above single factor experiments in ultrapreus water, the best conditions for hydrogel preparation were obtained as follows: 0.07wt% of cross-linker BAC, 4:2 molar ratio of HEAA to AA, 1.25wt% initiator APS, and 70% neutralization of acrylic acid.

References

[1] X. Zhou, Y. Ma, N. Wang, Z. Lei, *Materials Today Communications.* **2024**, *39*, 109190.

[2] A. Olad, H. Zebhi, D. Salari, A. Mirmohseni, A. Reyhanitabar, *Journal of Porous Materials.* **2018**, *25*, 665.

[3] Y. Li, X. Li, C. Chen, D. Zhao, Z. Su, G. Ma, R. Yu, *Carbohydrate Polymers.* **2016**, *151*, 1251.

[4] T. Chen, H. Liu, C. Dong, Y. An, J. Liu, J. Li, X. Li, C. Si, M. Zhang, *Carbohydrate polymers.* **2020**, *247*, 116717.

[5] M. Ramazani-Harandi, M. Zohuriaan-Mehr, A. Yousefi, A. Ershad-Langroudi, K. Kabiri, *Journal of applied polymer science.* **2009**, *113*, 3676.

[6] M. Chen, Y. Shen, L. Xu, G. Xiang, Z. Ni, *RSC advances.* **2020**, *10*, 41022.

[7] X. Liu, S. Cheng, J. Zhao, X. Qiu, W. Zhang, G. Ma, Z. Lei, *Journal of Applied Polymer Science.* **2021**, *138*, 50103.
